# Recent Advances in Caries Prevention and Non-Invasive Management: From Ecological Modulation to Precision Interventions

**DOI:** 10.3390/dj14090555

**Published:** 2026-09-02

**Authors:** Yanfei Yin, Wenxia Li, Wei Zhang, Ying Huang, Lingying Fan, Xiaolin Wei, Dongyang Ma

**Affiliations:** 1 Healthy Examination & Management Center, Second Hospital of Lanzhou University, Lanzhou 730000, China; yinyf2025@lzu.edu.cn (Y.Y.); 18368913110@163.com (W.L.); zhangwei14@lzu.edu.cn (W.Z.); 13919818350@163.com (L.F.); wxl13008782830@163.com (X.W.); 2 Department of Stomatology, Second Hospital of Lanzhou University, Lanzhou 730000, China; huangying2025@lzu.edu.cn

**Keywords:** caries prevention, probiotics, nanotechnology, remineralization, precision risk assessment

## Abstract

Background: Dental caries remains a globally prevalent biofilm-mediated disease driven by microbial dysbiosis and progressive enamel demineralization. The traditional “drill and fill” paradigm has increasingly given way to biologically informed preventive frameworks emphasizing ecological management. Methods: This narrative review synthesizes 134 studies (2016–2026) retrieved from PubMed, Web of Science, Scopus, and Google Scholar to evaluate emerging caries prevention strategies across three domains. Results: Three interrelated domains of advancement were identified. First, microbial ecological modulation—including probiotics, prebiotics, and targeted antimicrobial peptides—aims to restore oral microbiome homeostasis by disrupting cariogenic virulence without eliminating commensal flora. Second, nanomaterial-based targeted interventions utilizing stimuli-responsive carriers and biofilm-penetrating nanoparticles enable site-specific drug delivery with enhanced therapeutic efficacy. Third, precision-guided strategies incorporating caries risk assessment models and salivary biomarker diagnostics facilitate individualized prevention protocols. Conclusions: These findings demonstrate a fundamental paradigm shift from indiscriminate microbial eradication to equilibrium-driven, ecology-centered disease management. Future directions include AI-assisted personalized prevention, chairside microbiome diagnostics, and smart responsive biomaterials for sustained caries control.

## 1. Introduction: Evolution of Dental Caries Prevention and Treatment Strategies

Dental caries is a biofilm-mediated, diet-modulated, sugar-driven, multifactorial, dynamic, non-communicable disease influenced by biological, behavioral, psychosocial, and environmental factors. Dental caries continues to impose a substantial global burden, and the number of cases of untreated caries in both permanent and deciduous teeth has increased over recent decades [1]. Caries causes pain and infection, impairs productivity, and shares common risk factors—particularly high sugar intake and socioeconomic disadvantage—with metabolic diseases such as diabetes and cardiovascular disease [2]; however, current evidence for a direct causal link remains limited and does not yet warrant modifications to clinical practice guidelines.

The oral cavity is a complex microbial ecosystem containing approximately 700–1000 microbial species [3]. Specific microorganisms and ecological imbalance of the oral microbiota are involved in the pathogenesis of caries [4]. Oral bacteria metabolize dietary carbohydrates, thereby disrupting the balance between remineralization and demineralization processes and causing damage to the dental structure [5]. Improved understanding of the ecological mechanisms underlying caries development has shifted prevention strategies from the elimination of cariogenic bacteria to ecological regulation and from passive restoration to active intervention. This narrative review synthesizes and critically appraises recent developments in caries prevention, with an emphasis on three major emerging strategies: microbial ecological regulation, targeted interventions, and precision-guided supportive strategies.

The literature search for this review was primarily conducted through major academic databases such as PubMed, Web of Science, Scopus, and Google Scholar, focusing on the period from 2016 to 2026 to cover the latest advancements in caries prevention and non-invasive management. Search keywords included, but were not limited to, “Caries prevention”, “Microecological modulation”, “Targeted interventions”, “Precision risk assessment”, “Probiotics”, “Nanotechnology”, and “Remineralization”, as well as their synonyms and combinations of related terms. An initial search identified a large number of relevant publications. After preliminary screening of titles and abstracts, irrelevant topics, non-English literature, and duplicate entries were excluded. Subsequently, the full texts of the remaining articles were thoroughly evaluated to ensure relevance to the three major emerging strategies: microbial ecological modulation, targeted interventions, and precision-guided supportive strategies. Ultimately, 134 publications, including reviews, original studies, and clinical trials, were included, providing a comprehensive and critical appraisal of recent developments in this field.

## 2. Microecological Regulation: Restoration of Oral Health Balance

**Figure 1:**
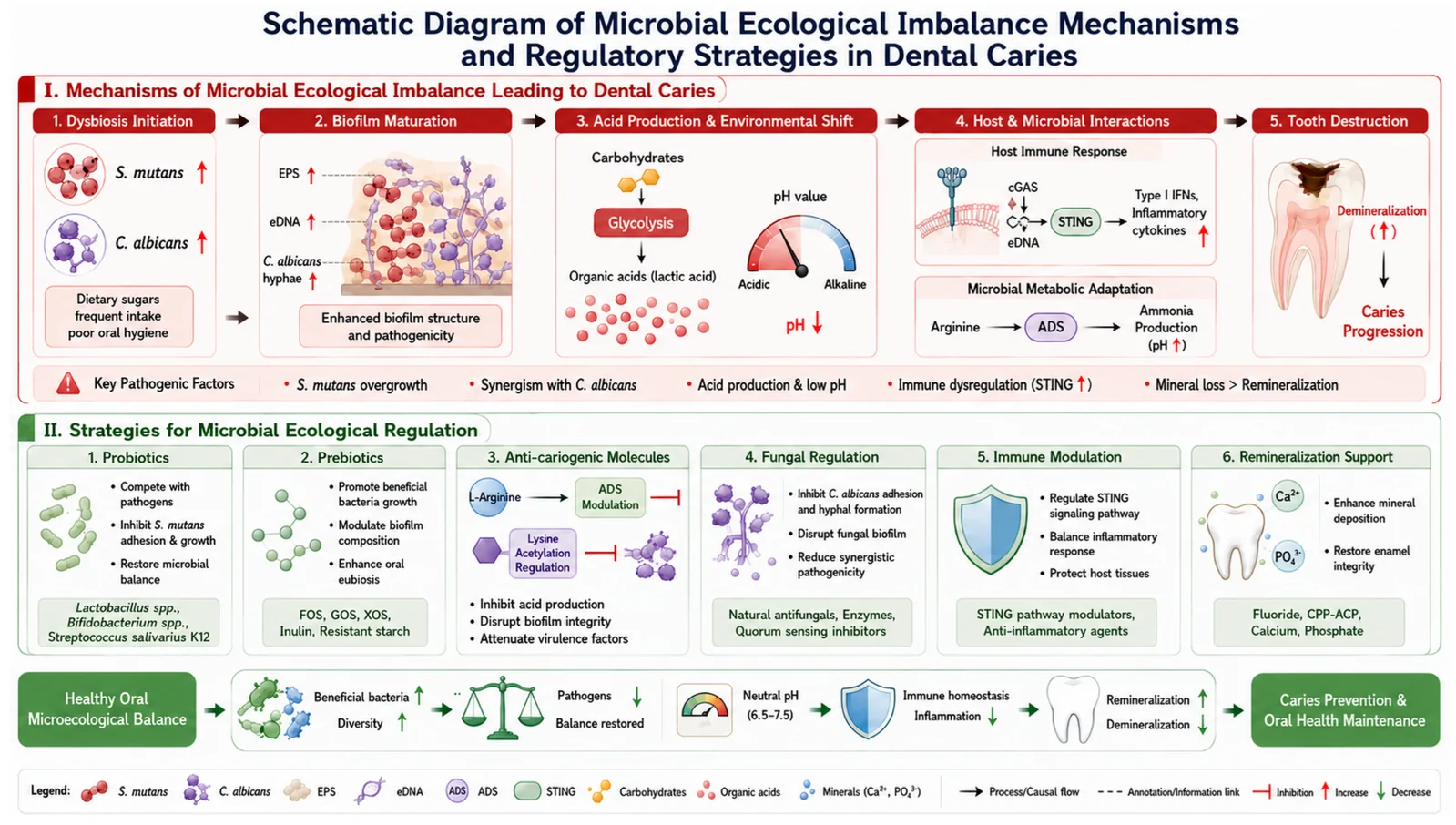
Mechanisms of microbial ecological imbalance in dental caries and strategies for regulating microbial ecology (this is an original schematic diagram scientifically integrated and simplified based on Section 2 “Microecological Regulation: Restoration of Oral Health Balance”). Arrows indicate process/causal flow, annotation/information links, inhibition, increase, or decrease, as defined in the figure legend.

Microbial imbalance is considered a primary cause of dental caries [4]. Therefore, restoring this imbalance is crucial for both the prevention and management of dental caries. Microecological regulation aims to restore the ecological balance of the oral microbiota through various approaches. The principal mechanisms of microbial ecological imbalance and the corresponding regulatory strategies are summarized in Figure 1.

### 2.1. Probiotic and Prebiotic Therapy

Probiotic therapy has emerged as a promising strategy for the prevention and control of dental caries through oral microecological modulation [6,7]. The primary mechanisms include (1) inhibiting the adhesion and proliferation of cariogenic bacteria; (2) competitively occupying binding sites on the oral mucosa and tooth surfaces [8,9]; (3) degrading pathogenic biofilms through enzymatic activity; (4) regulating oral pH through arginine metabolism; and (5) modulating oral immune responses to enhance the host’s defense against pathogens [10,11,12,13]. Nizami et al. [14] carried out a systematic review of the mechanisms and clinical effectiveness of probiotics in caries prevention, highlighting that certain strains, such as *Lactobacillus reuteri*, *Lactobacillus rhamnosus* and *Streptococcus salivarius*, showed beneficial effects in inhibiting cariogenic bacteria through competitive exclusion, alkali production via arginine metabolism, and the production of antimicrobial substances such as reuterin and bacteriocins. In addition, *Lactobacillus salivarius* supernatant has been shown to inhibit the growth of *Streptococcus mutans* biofilms at different time points [15]. Clinical trials have reported reductions in salivary *S. mutans* counts with selected strains; however, meta-analytic evidence indicates that this surrogate outcome does not consistently translate into statistically significant reductions in caries incidence (OR 0.60, 95% CI crossing unity) [10]. Although selected probiotic strains significantly reduce salivary *S. mutans* counts in specific age groups, evidence for clinically meaningful reductions in caries incidence remains limited and heterogeneous, particularly in adults, for whom data are scarce.

In particular, probiotic therapy has shown preliminary signals of efficacy in select pediatric populations (e.g., *L. rhamnosus* SP1 reducing lesion progression over 10 months [16]), but effect sizes are small to modest and confidence intervals often include the null [17]. Therefore, probiotics represent a promising but incompletely validated complementary approach whose clinical benefit appears strain-specific, dose-dependent, and population-contingent.

Current challenges include strain selection and optimization, delivery methods, and maintenance of efficacy during long-term use. In addition, variability in study design, probiotic strains, dosages, and treatment duration makes standardization of probiotic therapy difficult [14]. Although probiotics and prebiotics exert their effects through modulation of the oral microbial ecology and inhibition of acid production, they cannot replace fluoride as the foundation of caries prevention. Nevertheless, the integration of probiotics into personalized oral health care strategies, shifting from the traditional “targeted eradication of cariogenic bacteria” to “restoration of microbial balance” represents a promising paradigm shift in caries prevention.

### 2.2. Novel Anti-Cariogenic Molecules: Targeting Microbial Interactions

L-Arginine: Arginine is a semi-essential amino acid that plays important roles in protein synthesis, nutrient metabolism, immune regulation, and anti-inflammatory and antioxidant activities, in addition to participating in various signaling pathways [17]. L-Arginine is metabolized by commensal bacteria via the arginine deiminase system (ADS) to produce ammonia, contributing to biofilm pH homeostasis. In vitro studies using exogenous L-arginine (typically 1.5–8% *w*/*v*) have demonstrated inhibition of *S. mutans* planktonic growth and biofilm formation, suppression of extracellular polysaccharide (EPS) production, and modulation of microbial community composition favoring health-associated species. However, clinical translation remains uncertain: a systematic review found that evidence for arginine–fluoride toothpaste superiority is subject to high risk of bias and conflicts of interest, precluding firm conclusions [18].

Gao et al. [19] investigated the regulatory effects of L-arginine on two of the most important cariogenic microorganisms, namely *S. mutans* and *C. albicans*. Using assays for planktonic growth measurement, biofilm biomass analysis, crystal violet staining, colony-forming unit (CFU) counting, extracellular polysaccharide (EPS) production, and lactic acid production in the dual-bacterial biofilm, the authors demonstrated that L-arginine inhibited both planktonic growth and biofilm formation in single and mixed-species cultures. In addition, L-arginine reduced biofilm adhesion and significantly decreased extracellular polysaccharide production and acidogenic metabolism. These findings suggest that L-arginine may provide a promising strategy for disrupting cross-species microbial interactions and reducing their synergistic cariogenic effects.

Lysine acetylation has been identified as a widespread PTM in oral bacteria. In *S. mutans*, 973 acetylation sites across 445 proteins have been catalogued, with enrichment in metabolic and virulence-associated pathways. Related acylation modifications—including lysine lactylation (Kla) and succinylation (Ksuc)—have also been mapped. Notably, reduced lactylation of RNA polymerase subunit α at Lys173 under high-sugar conditions was associated with enhanced EPS synthesis, suggesting a regulatory link between metabolic state and biofilm virulence [20]. However, these findings derive from proteomic profiling and mutational analyses under controlled laboratory conditions. The directionality and causal relevance of acetylation to cariogenicity in vivo remain to be established, and no validated therapeutic targets have yet emerged from this work [21].

### 2.3. Role and Regulation of Fungi in Caries

For a long time, dental caries has been considered to be caused by acid-producing metabolism and bacterial biofilm formation by cariogenic bacteria such as *S. mutans*, and preventive and therapeutic approaches have been mainly oriented towards these bacteria. However, recent studies indicate that fungi, particularly *C. albicans*, play an important role in the pathogenesis and progression of dental caries, especially root caries [22]. *C. albicans* and *S. mutans* exhibit synergistic cariogenic effects and are frequently co-detected at high levels in various oral diseases, including early childhood dental caries [23,24], root caries [25], and denture stomatitis [26,27]. Increasing evidence suggests that cariogenic bacteria and fungi cooperate to form highly pathogenic biofilms [28,29]. The synergistic pathogenicity of *S. mutans* and *C. albicans* is mediated through enhanced adhesion, increased metabolic activity, acid production, and cross-feeding interactions [30,31]. These microorganisms produce extracellular polysaccharides that facilitate the formation of complex cross-species biofilms. In addition, their interactions promote carbohydrate metabolism, upregulation of virulence-related genes, biomass accumulation, enhanced metabolic activity, and reduced pH, thereby intensifying the pathogenicity of the biofilm [32,33].

Recent mechanistic hypotheses propose that fungal biofilm-derived extracellular DNA (eDNA) and DNA-carrying extracellular vesicles (EVs), together with bacterial eDNA, constitute structural components of the cariogenic biofilm matrix and may serve as pathogen-associated molecular patterns (PAMPs) capable of activating the host cGAS-STING signaling pathway [34]. This hypothesis draws on experimental evidence from systemic candidiasis models showing that *C. albicans* biofilm-associated DNA packaged in EVs triggers cGAS-STING-dependent type I IFN signaling [35]. However, whether this pathway is functionally relevant in the specific microenvironment of dental caries—where hard tissue demineralization rather than soft tissue inflammation is the primary outcome—remains to be experimentally validated in caries-specific in vivo models. The potential dual role of STING activation (protective antimicrobial defense vs. tissue-damaging inflammation) further complicates interpretation and warrants caution in extrapolating from non-caries contexts.

## 3. Targeted Intervention: Precision Therapy and Repair

**Figure 2:**
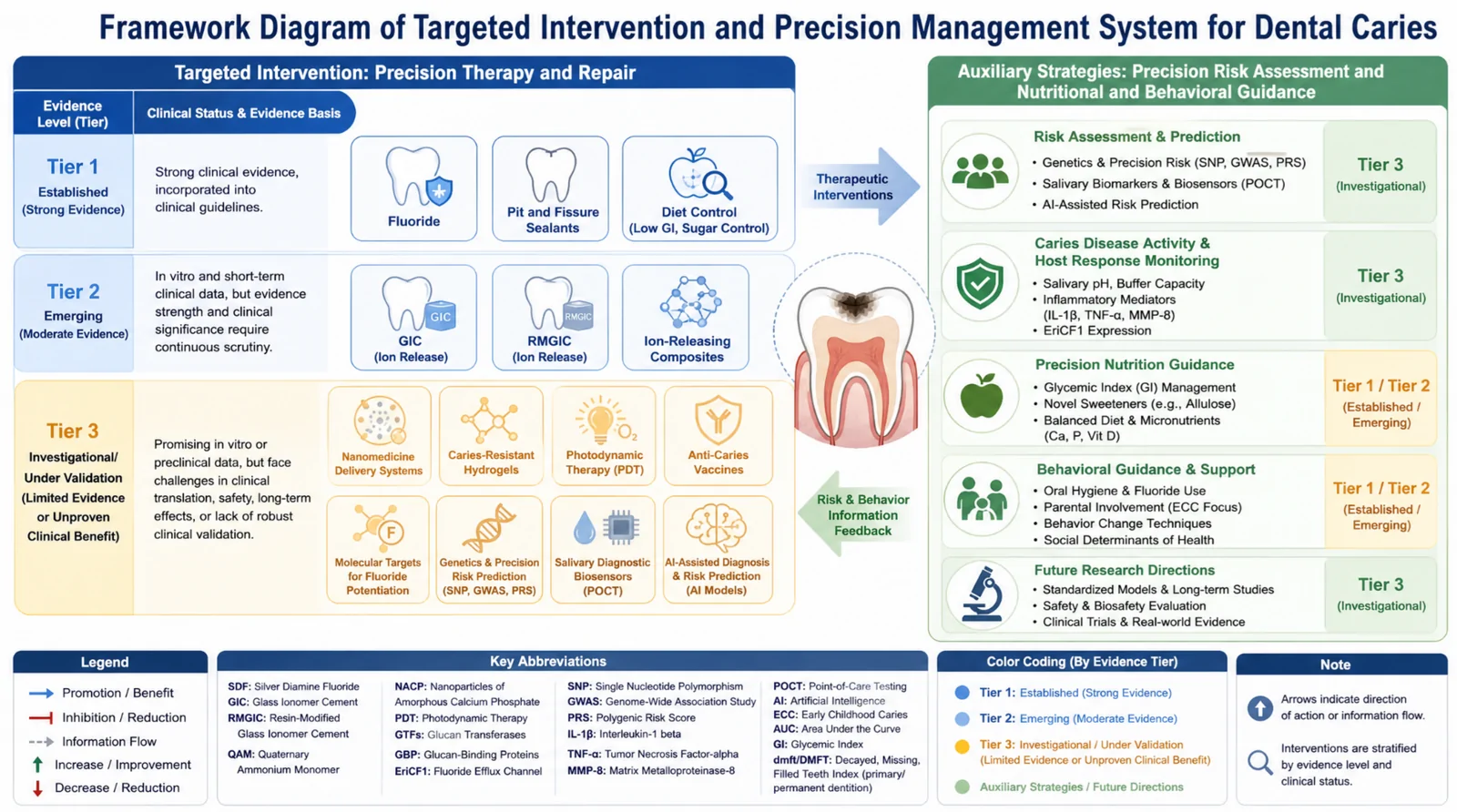
Targeted intervention and precision management system for dental caries (this is an original schematic diagram scientifically integrated and simplified based on Section 3 “Targeted Intervention: Precision Therapy and Repair” and Section 4 “Auxiliary Strategies: Precision Risk Assessment and Nutritional Behavior Guidance”).

Beyond microecological regulation, innovative intelligent materials and molecular-targeted interventions provide further avenues for the precise treatment and restoration of dental caries. An overview of the targeted interventions and complementary precision-guidance strategies discussed in Section 3 and Section 4 is presented in Figure 2.

### 3.1. Intelligent Materials and Nanomedicine Delivery Systems

Bioactive Materials: Bioactive restorative materials for caries management can be broadly classified as (a) fluoride-based materials (e.g., SDF), (b) calcium- and phosphate-based materials, (c) bioactive glass-containing composites, (d) metal- or metal-oxide-based nanomaterials, and (e) peptide-based materials [36]. However, there is no universally accepted definition of “bioactive” in cariology; the term is generally applied to materials that can promote hydroxyapatite formation on tooth surfaces or release therapeutically active ions [36]. Glass ionomer cements have the longest clinical track record. In an in vitro biofilm model, both conventional and resin-modified GICs inhibited secondary caries adjacent to restorations compared with bonded resin composites [37]. A composite containing 5% DMAHDM and nanoparticles of amorphous calcium phosphate (NACP) reduced cariogenic bacterial counts by 2–5 log units while maintaining acceptable flexural strength; when the NACP content was increased to 30%, enamel hardness at restoration margins doubled under biofilm acid challenge [38]. Novel bioactive resin composites combining remineralizing and antibacterial functions have also shown promise in inhibiting secondary caries in enamel and dentin under oral biofilm conditions [39]. Nevertheless, most of this evidence remains in vitro, and an in situ systematic review found insufficient evidence to rank the caries risk associated with eight restorative material types because of substantial heterogeneity [40].

A recent meta-analysis pooling 40 RCTs reported that bioactive restorative materials collectively reduced secondary caries risk by 45% relative to conventional materials [41]. However, this aggregate estimate requires careful contextualization. (1) Effect measure and precision: The specific effect measure (RR, OR, or HR), confidence interval, and heterogeneity statistics (I2, τ2) should be reported to assess estimate stability. A separate meta-analysis of GIC versus amalgam found RR = 0.55 (primary teeth) and RR = 0.20 (permanent teeth), but GIC versus resin composite showed no significant difference [42]. (2) Material heterogeneity: The “bioactive” category encompasses GIC, RMGIC, ion-releasing composites, and contact–kill QAM-containing resins—materials operating through fundamentally different mechanisms. A network meta-analysis of 50 RCTs identified that adhesive strategy interacts significantly with material type [43], while Albelasy et al. found no significant difference between ion-releasing restorations and resin composite in either secondary caries or marginal adaptation [44]. (3) Analysis unit and clustering: Whether the unit was the patient, tooth, or restoration and whether within-patient clustering was addressed significantly affect effect size and confidence intervals. (4) Follow-up duration: Pinto et al. demonstrated that material ranking changes between 1-year and ≥2-year follow-up [45], suggesting that short-term results may not predict long-term efficacy. The “45% reduction” should therefore be interpreted as a heterogeneous pooled estimate across mechanistically distinct material classes, not as a uniform therapeutic effect of any single product category.

The oral delivery of nanomedicines is often ineffective for preventing and treating caries due to poor tissue penetration, short duration of action, and lack of targeted delivery. In addition, some chemical agents exhibit dose-dependent side effects, and the emergence of drug-resistant bacterial strains is further reducing the therapeutic value of current treatments [46].

In this scenario, nanomaterials present innovative solutions due to their superior antibacterial properties, excellent biofilm penetration, targeted delivery capabilities, and adjustable physicochemical characteristics, making them promising for combating caries-causing bacteria and regulating biofilms [47,48]. Nanomedicine delivery systems for caries management exploit particle sizes of 1–100 nm to enhance tissue penetration and enable targeted drug release at lesion sites [49]. However, the performance of different nanoplatforms varies substantially. Common carriers include liposomes, solid lipid nanoparticles, inorganic nanoparticles (such as silica, gold, and silver), polymer nanoparticles, and polymer micelles [48]. Du et al. [50] summarized the progress made in the development of such systems for the treatment of caries with the potential to greatly enhance the stability, solubility, and bioavailability of drugs and reduce systemic side effects. The mechanisms of action are inhibition of bacterial activity, prevention of biofilm formation, slowing dental demineralization, and promoting remineralization, making their use a very important caries prevention and management strategy. The clinical translation of nanomedicine systems for oral use requires addressing several unresolved concerns. Predictive safety assessment frameworks emphasize the need for structure–activity relationships to anticipate nanoparticle toxicity prior to clinical testing [51]. Specific challenges in the oral context include (1) chronic low-dose exposure: daily or repeated topical application creates cumulative exposure patterns distinct from single-dose systemic administration; (2) inadvertent ingestion: oral nanomaterials face inevitable gastrointestinal exposure through swallowing, requiring systemic toxicity data; (3) microbiome selectivity: broad-spectrum antibacterial nanomaterials may disrupt commensal oral flora beyond target pathogens, potentially selecting for resistant strains; (4) environmental release: antimicrobial nanomaterials entering wastewater via oral rinsing may contribute to environmental resistance selection; and (5) nanoparticle accumulation: potential deposition in periodontal tissues or dental tubules may have unknown long-term biological consequences. Currently, ferumoxytol represents a unique case as an FDA-approved nanoparticle formulation (originally for systemic iron deficiency) that has been repurposed for topical oral biofilm disruption with demonstrated in vivo efficacy in animal models [52]. No other oral anti-caries nanoplatform has achieved regulatory approval.

Multifunctional Anti-Cariogenic Nanomaterials: This concept addresses the recognized limitation that single-function materials cannot simultaneously achieve early detection, biofilm disruption, and hard tissue repair. While review articles have categorized desired functionalities (early diagnostics, mechanochemical biofilm removal, simultaneous anti-biofilm, and remineralization delivery), original experimental validation of integrated multifunctional systems remains limited. A notable exception is the dental-plaque-inspired nanosystem developed by Xu et al., which combines self-assembling peptide (SAP) conjugation for enamel adhesion with pH-triggered release of both antibacterial and restorative agents in the caries-specific acidic microenvironment [53]. This system illustrates the proof-of-concept stage of multifunctional integration. Critical gaps remain: (1) no system has demonstrated simultaneous diagnostic + therapeutic function in vivo; (2) the added complexity increases manufacturing challenges and regulatory barriers; and (3) long-term stability and safety of multi-component nanosystems in the oral environment are untested.

### 3.2. Caries-Resistant Hydrogel

The 3D network structure of hydrogels gives them properties like conductivity, expansibility, environmental sensitivity, and viscosity [54,55,56,57,58,59]. These properties provide a considerable advantage to the activity and action time of the agents used for caries prevention, as they enhance their duration and retention in the target site [60]. Hydrogels are also highly hydrophilic and stable in water for long periods of time after absorbing water [61]. This stability is much greater than that of conventional materials, adding to the length of time that the agent acts and its ability to stay on the target site [62]. In addition, the 3D structure of crosslinked polymers of hydrogels enables the effective incorporation of active ingredients and can act as a scaffold for remineralization [63]. Hydrogels are a promising material for caries management due to these properties. The use of traditional materials and hydrogels together can have a great impact on the effectiveness of caries prevention and treatment [64,65,66,67,68,69]. A hydrogel is a basic composition comprising a main matrix, which acts as a carrier, and functional components that give it some functionalities. The matrix, which is usually made of natural, synthetic, or semi-synthetic polymers, is used as a carrier of the active substance(s) [70,71]. These loaded components have a variety of functions, including antibiotic activity, remineralization, delivery of probiotics, and regulation of saliva. The hydrogels used for prevention and treatment of caries are mostly natural or semi-synthetic. Chen et al. [72] reviewed research advances on various categories of caries-preventive hydrogels, namely probiotic-loaded, antibacterial, remineralization-inducing, and saliva-regulating hydrogels, in a systematic review. Their mechanisms of action, functional effects, present research status, and limitations were analyzed in detail. In general, hydrogels have great application potential in managing oral microecological disorders, inducing remineralization of dental tissues, and providing specific caries prevention, and they can be used as a platform for the creation of multifunctional and precise caries intervention methods.

However, the current field of caries-resistant hydrogels is beset by several systemic deficiencies that constrain reliable appraisal of their clinical benefit. Future investigations should address the following improvements: (1) adoption of standardized multispecies biofilm models for efficacy evaluation to enhance clinical predictiveness [73]; (2) complete reporting of all critical experimental parameters—particularly those pertaining to phototherapy (photosensitizer dose, light wavelength, irradiance, exposure time, penetration depth, lesion location, and comparator)—to strengthen inter-study comparability and reproducibility [74]; (3) systematic evaluation of pulpal safety, selectivity toward commensal flora, and long-term microecological impact [75]; (4) explicit stratification of human randomized controlled trial (RCT) evidence from in vitro and animal experimental data in research reporting; and (5) design of longitudinal studies with extended follow-up periods to evaluate long-term caries-preventive efficacy and potential adverse events [76]. Only by systematically bridging these evidence gaps can caries-resistant hydrogels advance from the proof-of-concept stage to evidence-based clinical practice.

### 3.3. Photosensitive Nanomaterials and Photodynamic Therapy

Photodynamic Therapy (PDT) utilizes photosensitizers to produce reactive oxygen species under specific light excitation, selectively targeting cariogenic bacteria through photoactivation, making it a promising antibacterial intervention with broad applications [77]. Curcumin, known for its diverse biological activities, such as antibacterial [78], antifungal [79], anti-inflammatory [80], antitumor [81], and antioxidant [82] effects, is widely used as a natural photosensitizer. In PDT, curcumin shows high cytotoxicity against pathogenic microorganisms, especially Gram-positive bacteria [83]. Nanotechnology enhances its therapeutic potential by improving curcumin’s stability, bioavailability, and antibacterial efficacy [84,85,86]. Erckmann et al. [87] developed zinc-based nanocapsules containing curcumin (nano-curcumin) using a nanosuspension precipitation method and assessed their in vitro antibacterial effects on dental enamel samples colonized with *S. mutans* under both photodynamic-activated and non-activated conditions. The results showed nearly 100% encapsulation efficiency, uniform morphology, excellent dispersion, sustained curcumin release for up to 24 h, and superior cellular compatibility. Nano-curcumin significantly reduced bacterial colony-forming units (CFU), with the most pronounced effect in the photodynamic-activated group, indicating its potential in minimally invasive caries treatment. This strategy combines natural photosensitizers with biodegradable polymer carriers, offering a safer and less invasive therapeutic approach for clinical scenarios like pediatric dental caries. The design of multifunctional nanoplatforms further broadens the scope of PDT. Despite promising preclinical studies, several challenges must be addressed before photosensitive nanomaterial-mediated PDT can be widely adopted. The complex anatomy of the oral cavity and saliva flow challenge the retention time and targeting efficiency of nanomaterials. Different depths of carious lesions require varying light penetration capabilities, necessitating optimization of light source parameters for effective activation of deep lesions. Long-term biosafety concerns, including the metabolic pathways of nanomaterials and potential cytotoxicity, require rigorous evaluation. Finally, the lack of standardized treatment protocols, such as optimal photosensitizer concentration, irradiation dose, and exposure duration, hinders clinical translation.

### 3.4. Caries Prevention Vaccine

Vaccines are biological products that elicit specific immune responses to diseases by inducing antibody production and activation of immune mechanisms. Components on the surface of *S. mutans*, like glucan transferases (GTFs) and glucan-binding proteins (GBP), have previously been explored in vaccine development. Kazerooni and Hemmati reviewed cytokines, chemokines, and immune cells involved in oral immunity toward *S. mutans*, describing the roles of Th1, Th2, and Th17 responses as well as the suppressor of the cytokine signaling (SOCS) pathway in modulating vaccine-induced immunity [88]. Anti-caries vaccine strategies ideally aim to skew responses toward Th2/Th17 to promote salivary sIgA, not simultaneously suppress and induce Th1. The ERK/MAPK, NF-κB, and inflammasome pathways are involved in innate immune activation downstream of pathogen recognition, but their role as vaccine targets (rather than descriptors of infection pathology) requires cautious interpretation. The suggestion to “inhibit SOCS gene activation” to enhance vaccine efficacy is mechanistically plausible (as SOCS proteins negatively regulate JAK-STAT signaling) but remains an in vitro observation without clinical validation. Other vaccines, such as “Formalin-inactivated donor strain 2-recombinant protein antigen c” and “anti-CAT-SYIIgY antibody,” have had success in suppressing colonization with *Streptococcus mutans*, thereby reducing the risk of caries, the burden of disease, and health care costs [89]. Another important area of vaccine platform technology is DNA vaccines, and an innovative approach is edible vaccines based on nanodelivery. The results of animal studies suggest that caries-preventive vaccines may be developed for human application, but there are challenges. The oral mucosa immune environment is immunologically tolerant, and saliva flow decreases the concentration of antibodies, creating difficulties for long-term local immune responses. Furthermore, the pathogenesis of caries is not just a disease caused by *S. mutans* but a multimicrobial disease that reduces the effectiveness of single-antigen vaccines. Furthermore, there is no universally agreed upon correlate of protection, and it remains unclear what antibody levels and/or cellular immune response will provide reliable caries prevention in humans, which creates challenges when designing clinical trials and measuring endpoints. Researchers are exploiting their detailed knowledge of major virulence factors and advanced technologies, such as DNA-based vaccines, nanodelivery of vaccines, edible plant-based vaccines, and novel vaccine adjuvants, to enhance the efficacy and usability of candidate vaccines.

### 3.5. Molecular Targets for Fluoride Potentiation

Fluoride is widely used to prevent dental caries. Fluoride exerts its caries-preventive effect principally by reducing demineralization and promoting remineralization of dental hard tissues by forming fluorapatite, which is more resistant to acid dissolution (critical pH lowered to approximately 4.5 compared to 5.5 for hydroxyapatite). But, it is insufficient on its own to control the primary cariogenic bacterium *S. mutans*. Many microorganisms possess membrane proteins that maintain normal growth by expelling excess F^−^ ions [90]. Research has shown that *S. mutans* employs a fluoride efflux mechanism, actively pumping fluoride ions out through the EriCF1 channel, which reduces fluoride’s bacteriostatic effectiveness [91,92]. This fluoride efflux mechanism decreases the bacteriostatic effect of fluoride. To address this, three derivatives of diphenylurea were screened using molecular docking against the EriCF1 channel. Checkerboard dilution assays demonstrated that these compounds potently inhibit fluoride efflux, rendering *S. mutans* highly susceptible to sodium fluoride (NaF). The compound also exhibited favorable in vivo antibacterial synergy and biocompatibility [93].

The study identifies a promising intervention strategy through fluoride efflux pumps and provides novel insight into precision caries prevention. Future strategies could consist of a combination of “fluoride + synergist” treatment that will decrease the amount of fluoride required and increase caries prevention efficacy. The strategy does not currently constitute a validated precision prevention approach, and claims regarding reduced fluoride requirements or enhanced caries control efficacy await extensive preclinical characterization and human clinical validation. Fluoride exerts its effects at multiple levels, modifying the crystal structure at the molecular level and contributing to public health initiatives at the population level. The authors propose several future directions, including improving the accuracy of risk assessment tools, optimizing treatment protocols, and investigating synergistic interactions between fluoride and other bioactive materials to achieve more efficient and safer caries control.

## 4. Auxiliary Strategies: Precision Risk Assessment and Nutritional Behavior Guidance

Alongside microecological regulation and targeted interventions, precise risk assessment and personalized nutritional and behavioral guidance are essential for preventing dental caries.

### 4.1. Precision Risk Assessment

Genetics and Precision Risk Prediction: Individual susceptibility to dental caries varies considerably, with genetics contributing meaningfully to phenotypic variation. Twin studies have reported higher concordance in caries experience among monozygotic compared to dizygotic twins, supporting a heritable component [94]. Dental enamel, while mechanically resilient, lacks regenerative capacity; caries therefore arises most readily when genetic predisposition interacts with environmental exposures [95,96,97]. Single nucleotide polymorphisms (SNPs) have been implicated in modulating this susceptibility [98,99]—for example, the rs6656267 locus in ACTN2 and rs7209537 in LPO have been statistically associated with caries in candidate gene studies [100]. Proposed biological pathways include enamel development, salivary composition, and immune modulation [101]. However, several critical methodological caveats must be acknowledged. Traditional candidate gene studies are prone to non-replication, typically report small effect sizes, and are often ancestry-dependent. The field has accordingly shifted toward genome-wide approaches: consortium-based GWAS meta-analyses involving up to 19,003 children [96] and combined clinical/self-report datasets identifying 47 novel risk loci [102] have advanced understanding of the polygenic architecture. A meta-analysis of enamel formation genes has confirmed specific variants such as rs17878486 in AMELX (OR = 1.40) [103], and transcriptome-wide approaches have linked bitter taste receptor genes (TAS2R43, TAS2R14) to early childhood caries [104]. Nevertheless, current polygenic risk scores (PRS) demonstrate limited discriminative ability in general populations, and their clinical utility remains unestablished. PRS may hold value primarily in early disease stages to assist diagnosis or inform treatment choices [105], but without replicated effect sizes, external validation across diverse ancestries, and demonstrated predictive performance beyond existing clinical tools, the cited genetic variants should not be considered clinically actionable at present. Pang et al. constructed a machine learning caries risk prediction model incorporating SNPs and environmental factors in 1055 teenagers followed for 21 months, achieving an AUC of 0.78 [106]. While this represents progress, external validation in independent populations is lacking. Achieving precise genetic risk prediction will require large-scale, multi-ethnic, phenotypically standardized cohorts, rigorous reporting of discrimination and calibration metrics [107], and integration with established clinical caries risk assessment frameworks to demonstrate incremental value [108].

Salivary Diagnostic Biosensors: Saliva offers an accessible, non-invasive diagnostic medium enriched with host-derived DNA, RNA, proteins, metabolites, and microbial information [109]. Biosensor technology pairing biological recognition elements with signal transduction systems enables rapid point-of-care detection of molecular markers. Critically, however, the proposed markers IL-1β, TNF-α, and MMP-8 are not caries-specific. These inflammatory mediators are strongly influenced by periodontal inflammation and systemic conditions. A systematic review of salivary biomarkers for periodontal disease found that IL-1β and TNF-α could not reliably differentiate periodontal health from disease in isolation, highlighting their lack of diagnostic specificity for any single oral condition. Adeoye and Su explicitly noted the “equivocal accuracy of salivary biomarkers during validation” across oral diseases [110]. Buzalaf et al. similarly recognized that while saliva reflects pathophysiological conditions, it serves as a shared diagnostic medium for caries, periodontitis, and cancer, without disease-specific discrimination being well-established [111]. More informative biomarker combinations have been explored. Random forest modeling of 44 candidate markers identified IL-4, IL-13, IL-2-RA, and eotaxin/CCL11—rather than the traditionally cited inflammatory markers—as most important for caries classification [112]. Salivary microbial biomarkers may assist in predicting future caries in caries-free 1-year-old children, while proteomic meta-analysis has confirmed potential for ECC detection. Nevertheless, robust caries risk testing requires standardized sampling times and inclusion of biomarkers from multiple functional categories (immune, adhesion, acid production) [113]. An ideal salivary diagnostic biosensor should constitute an integrated point-of-care testing (POCT) platform incorporating intelligent sampling, efficient sample pretreatment, multiplex detection, and wireless data transmission. Before clinical adoption, however, each proposed biomarker panel must undergo rigorous analytical validation (measurement accuracy), clinical validation (sensitivity and specificity against defined reference standards in adequately powered cohorts), and demonstration of clinical utility (evidence that test results change clinical decisions and improve patient outcomes).

Artificial Intelligence (AI): AI-assisted approaches have opened new possibilities for caries diagnosis and risk prediction [114]. Machine learning algorithms applied to ECC prediction have demonstrated high accuracy, sensitivity, specificity, and AUC values in systematic reviews [110], and AI models for caries detection on bitewing radiographs achieved pooled sensitivity of 0.87 and specificity of 0.89 [111]. Recent reviews suggest that AI methods may show superior specificity compared to traditional approaches, and hybrid frameworks integrating mathematical modeling with AI have been proposed for individualized caries risk estimation.

However, the current evidence base has substantial limitations that preclude clinical translation. First, numerous caries risk assessment tools already exist (Cariogram, CAMBRA, PreViser, NUS-CRA), yet most are not routinely used in practice or used to influence treatment decisions; AI tools must demonstrate clear incremental value over these established systems. Second, evidence suggests a pressing need to improve methodological standards: desirable models should not only discriminate between patients who will and will not develop disease but also provide accurate absolute risk estimation (calibration) [112]—a metric frequently ignored in current assessments [113]. Third, a 2025 systematic review of caries risk prediction models in children emphasized the need to prioritize external validation to ensure reliable and generalizable clinical predictions. A 2026 scoping review similarly called for standardizing validation protocols in AI-integrated caries detection. The principal caries risk assessment technologies and their validation requirements are summarized in Table 1.

### 4.2. Precision Nutrition and Behavioral Guidance

Diet and Caries Prevention: Nutritional factors are central to the complex etiological network of dental caries, involving interactions among oral microorganisms, host factors, and diet. A systematic review conducted according to PRISMA guidelines and the PICO methodology [115] demonstrated a strong association between diet and the epidemiology, pathophysiology, and prevention of dental caries in children and adolescents, emphasizing community-based interventions combined with policy-level changes to reduce oral health disparities. Dietary recommendations for caries prevention should align with established oral health guidance—principally by reducing the frequency and amount of fermentable carbohydrate exposure—rather than relying on the glycemic index (GI) as a proxy for cariogenic risk [116].

Allulose: Han et al. [117] examined the cariogenic potential of allulose using single-species, dual-species (*S. mutans* and *S. oralis*), and saliva-derived microcosm biofilm models. Allulose inhibited bacterial growth, acid production, and biofilm formation compared with sucrose and glucose, with effects comparable to xylitol and erythritol. Allulose-grown biofilms lacked dense extracellular polysaccharide structures, and saliva-derived microcosms maintained higher microbial diversity. The U.S. FDA has concluded that allulose does not promote dental caries based on its low cariogenic potential, and in vitro pH telemetry confirms that allulose does not produce sustained acid drops below the critical pH of 5.5. D-allulose has a low glycemic index and non-cariogenic properties that position it as a functional rare sugar. However, no published human clinical trials have assessed caries outcomes (dmft/DMFT increments) as primary endpoints following allulose consumption.

Behavioral Guidance: Effective caries prevention through dietary and behavioral change: (1) Adherence and motivation: Knowledge alone does not change behavior. Theory-based approaches (e.g., motivational interviewing, self-regulation strategies) and family-centered behavioral interventions targeting underlying behavioral causes of ECC are needed [118]. Mothers’ decisions regarding children’s cariogenic food exposure are influenced by complex family dynamics, including interaction frequency and relationship strength [119]. (2) Caregiver involvement: For pediatric populations, parental engagement is paramount. Family-centered programs such as the MySmileBuddy intervention target high-risk preschoolers and their parents through behaviorally focused approaches [118]. (3) Social determinants, commercial determinants, and health equity: Oral health disparities are driven by income, access, education, and the food environment. Effective oral health promotion requires addressing shared risk factors (sugar, tobacco) through comprehensive strategies ranging from individual counseling to broad social policy. Collaboration between dietary and dental professionals is recommended but inconsistently practiced. (4) Health literacy and feasibility: Interventions must be accessible across literacy levels and cultural contexts. Currently, very few validated tools are available for clinicians to assess children’s diets in relation to caries risk in routine practice [120], highlighting the gap between precision nutrition aspirations and clinical feasibility.

Future dietary guidance for caries prevention should be grounded in established evidence (reducing fermentable carbohydrate frequency), clearly distinguish glycemic properties from cariogenic potential, avoid premature clinical claims for novel sweeteners lacking human trial evidence, and systematically address adherence, caregiver engagement, social and commercial determinants, health literacy, and oral health inequities.

## 5. Conclusions and Prospects

Caries prevention is undergoing a significant conceptual transformation. Etiological paradigms have evolved from viewing caries as an infectious disease caused by specific bacteria to current frameworks emphasizing a mixed bacterial–ecological approach as responsible for lesion initiation and pathogenesis [121]. The microecological theory redefines caries from an “infectious disease” to an “ecological imbalance disorder,” offering new perspectives for prevention, early intervention, and precision treatment. This paradigm shift has enabled a broader transition—from surgical excision of affected tissue toward preventive and minimal intervention dentistry approaches [122].

### 5.1. Evidence-Stratified Framework for Caries Prevention

#### 5.1.1. Tier 1: Guideline-Supported Interventions with Robust Clinical Evidence

These interventions are supported by high-quality randomized controlled trials and systematic reviews and are incorporated into clinical practice guidelines [123]. They include fluoride exposure (toothpaste, varnish, water fluoridation), with dose–response relationships confirmed by systematic reviews [124]; pit and fissure sealants for occlusal caries prevention [125]; dietary sugar reduction as a cornerstone of caries prevention; and regular mechanical plaque removal. Caries prevention has traditionally relied on these established measures—fluoride exposure, diet control, thorough oral hygiene, and antibacterial approaches—and viewing caries as a non-communicable disease does not disqualify these methods but brings them into a new ecological context [126]. School-based comprehensive caries prevention programs integrating these measures have demonstrated potential to reduce caries by addressing access inequities [127].

#### 5.1.2. Tier 2: Emerging Interventions Lacking Adequate Human Clinical Evidence

Several ecological preventive approaches have been developed that could potentially broaden the arsenal of currently available caries-preventive measures [75]. These include probiotics and prebiotics for biofilm engineering early in life to support microbial diversity; arginine-containing formulations showing superior caries prevention in studies with high risk of bias [128]; novel anti-cariogenic molecules targeting virulence factors without affecting bacterial viability; and silver diamine fluoride for caries arrest in older adults, where limited studies support effectiveness beyond pediatric populations [129]. Methods to reduce dietary sugar intake, slow down plaque metabolism, and support saliva functions should be further developed and investigated in terms of efficacy, compliance, and cost-effectiveness. These approaches require well-designed randomized clinical trials with standardized caries outcomes before integration into routine clinical guidelines.

#### 5.1.3. Tier 3: Preclinical or Hypothesis-Generating Technologies

Technologies that remain at the preclinical stage or require substantial validation before clinical consideration include anti-caries vaccines, which have not advanced to late-stage clinical trials despite promising preclinical immunogenicity data; smart nanomaterial delivery systems and photosensitive nanomaterials for targeted biofilm disruption [130]; genomic and polygenic risk scores for caries prediction, whose clinical utility remains unestablished [105]; salivary biosensor platforms for real-time caries risk monitoring; and AI-integrated precision caries management systems. While AI-assisted caries diagnosis has shown accuracy in meta-analysis and machine learning models demonstrate promising performance metrics for early childhood caries prediction [131], external validation across diverse populations remains a critical unmet requirement. A comparative summary of these intervention categories, mechanisms, evidence certainty, limitations, and clinical status is provided in Table 2.

### 5.2. Limitations of This Review

Research heterogeneity: The included studies span diverse populations, age groups, outcome definitions, and follow-up periods. Inconsistent outcome reporting remains a significant hurdle to combining results across caries prevention trials [132], and CONSORT adherence in caries prevention RCTs remains suboptimal [133].

Reliance on secondary literature: Many conclusions are drawn from systematic reviews and meta-analyses rather than primary studies, introducing potential for information loss and inherited bias. Quality stratification in meta-analysis can itself induce collider stratification bias [134].

Predominance of preclinical evidence: For emerging interventions (nanomaterials, vaccines, novel ecological agents), the evidence base is dominated by in vitro and animal studies. Extrapolation to clinical effectiveness requires caution.

Potential publication and language bias: Despite efforts to include a diverse literature, English-language publications from high-income countries are over-represented. Studies from low- and middle-income countries—where caries burden is often greatest—may be underrepresented.

Rapidly evolving evidence: Given the pace of research in AI, genomics, and microbiome science, some conclusions may require revision as new evidence emerges.

### 5.3. Future Directions

Interdisciplinary collaboration remains essential for advancing caries prevention. However, future research should prioritize (1) pragmatic clinical trials comparing novel approaches against established preventive methods using patient-relevant outcomes; (2) standardized core outcome sets for caries prevention research; (3) external validation of prediction models in diverse populations; (4) implementation science addressing how evidence-based prevention can be delivered equitably at scale; and (5) health economic analyses to guide resource allocation. The integration of ecological modulation strategies with remineralization approaches represents a scientifically promising direction but must advance through rigorous evidence generation before claims of clinical utility can be substantiated. Strong evidence for caries primary and secondary prevention remains a compulsory foundation for clinical practice guideline development, and all emerging technologies must ultimately be held to this standard [123].

## Figures and Tables

**Table 1:** Caries Risk Assessment Technologies and Validation Requirements.

Technology Category	Key Features/ Mechanisms	Representative Markers/Methods	Current Status/Findings	Validation Requirements/Challenges	Evidence Level (Tier)
Genetics and Precision Risk Prediction	Individual susceptibility; heritable component	SNPs (e.g., ACTN2 and LPO) GWAS PRS	Evidence of genetic influence, but PRS has limited discriminative ability in general populations.	Large-scale, multi-ethnic cohorts Rigorous reporting of discrimination and calibration Integration with clinical frameworks External validation	Tier 3
Salivary Diagnostic Biosensors	Accessible, non-invasive diagnostic medium; rapid point-of-care detection	IL-1β, TNF-α, and MMP-8 (inflammatory mediators) IL-4, IL-13, IL-2RA, and eotaxin/CCL11 (more specific) Microbial biomarkers	Proposed markers often lack caries specificity More informative combinations explored Potential for ECC detection	Rigorous analytical validation (accuracy) Clinical validation (sensitivity/specificity against reference standards) Demonstration of clinical utility (changes clinical decisions)	Tier 3
Artificial Intelligence (AI)-Assisted Diagnosis and Risk Prediction	Machine-learning algorithms applied to ECC prediction and caries detection	AI models for ECC prediction AI for caries detection on radiographs	High accuracy, sensitivity, specificity, and AUC values in systematic reviews Superior specificity compared with traditional approaches	Demonstrate clear incremental value over existing tools Improve methodological standards (calibration) Prioritize external validation in diverse populations	Tier 3

*Abbreviations:* ACTN2, alpha-actinin-2; AI, artificial intelligence; AUC, area under the receiver operating characteristic curve; CCL11, C-C motif chemokine ligand 11; ECC, early childhood caries; GWAS, genome-wide association study; IL, interleukin; IL-2RA, interleukin-2 receptor alpha; LPO, lactoperoxidase; MMP-8, matrix metalloproteinase-8; PRS, polygenic risk score; SNPs, single-nucleotide polymorphisms; TNF-α, tumor necrosis factor alpha.

**Table 2:** Comprehensive Analysis of Caries Intervention Measures.

Intervention Category	Mechanism	Representative Evidence	Study Design	Outcomes	Limitations	Evidence Certainty	Clinical Status
Established Preventive Measures (Tier 1)							
Fluoride Exposure	Promotes remineralization, inhibits demineralization, suppresses bacterial activity	Toothpaste, varnish, water fluoridation	RCTs, systematic reviews	Significantly reduces caries incidence	–	High	Recommended in clinical guidelines
Pit and Fissure Sealants	Physical barrier, prevents bacterial invasion	Resin-based, glass ionomer	RCTs, systematic reviews	Effective in preventing occlusal caries	–	High	Recommended in clinical guidelines
Dietary Sugar Control	Reduces carbohydrate sources for cariogenic bacteria	Reduces frequency and quantity	Observational studies, systematic reviews	Lowers caries risk	Adherence challenges	Moderate	Recommended in clinical guidelines
Emerging Interventions (Tier 2)							
Probiotic/Prebiotic Therapy	Modulates oral microecology, inhibits cariogenic bacteria	*Lactobacillus rhamnosus* SP1	RCTs, systematic reviews	Reduces salivary *S. mutans* counts, partially reduces caries progression	Strain-specific, dose-dependent, heterogeneity, lack of adult data	Moderate	Promising, but requires more validation
Arginine Formulations	Produces alkaline substances via ADS, increases pH, inhibits biofilm	1.5–8% L-arginine	*In vitro* studies, systematic reviews	Inhibits *S. mutans* growth and biofilm formation	Clinical translation uncertain, systematic reviews with high risk of bias	Low	Still under investigation
Silver Diamine Fluoride (SDF)	Antibacterial, promotes remineralization, hardens carious lesions	–	Limited studies	Inhibits caries progression in older adults	Limited to specific populations, aesthetic concerns	Moderate	Clinical application in specific situations
Preclinical/Hypothesis-Generating Technologies (Tier 3)							
Novel Anti-Cariogenic Molecules	Targets virulence factors without affecting bacterial viability	Lysine acetylation regulation	*In vitro* studies	Affects bacterial metabolism and biofilm formation	Lacks *in vivo* and clinical validation	Very Low	Laboratory research stage
Nanomaterial Delivery Systems	Enhances tissue penetration, targeted drug release, multifunctional	Liposomes, inorganic nanoparticles, SAP nanosystems	*In vitro* studies, animal models	Antibacterial, biofilm disruption, hard tissue repair	Safety, long-term exposure, regulatory hurdles	Very Low	Laboratory research stage
Anti-Caries Hydrogels	Carrier function, scaffold role	Probiotic/antibacterial/remineralization-inducing hydrogels	*In vitro* studies	Extends duration of action, promotes remineralization	Lack of standardized models, long-term safety	Very Low	Laboratory research stage
Photodynamic Therapy (PDT)	Photosensitizers produce ROS and selectively target bacteria	Nano-curcumin	*In vitro* studies	Significantly reduces bacterial colony-forming units	Oral complexity, light penetration depth, biosafety	Very Low	Preclinical research stage
Anti-Caries Vaccines	Induces specific immune responses	Targets *S. mutans* GTFs/GBPs	Preclinical studies (animal)	Inhibits *S. mutans* colonization	Immune tolerance, multi-microbial pathogenesis, unclear correlates of protection	Very Low	Preclinical research stage
Molecular Targets for Fluoride Potentiation	Inhibits fluoride efflux pump	Diphenylurea derivatives	*In vitro* studies, *in vivo* studies	Enhances fluoride bacteriostatic effect	Lacks extensive preclinical and human clinical validation	Very Low	Laboratory research stage

*Abbreviations:* ADS, arginine deiminase system; GBPs, glucan-binding proteins; GTFs, glucosyltransferases; PDT, photodynamic therapy; RCTs, randomized controlled trials; ROS, reactive oxygen species; SAP, self-assembling peptide; SDF, silver diamine fluoride.

## Data Availability

No new data were created or analyzed in this study. Data sharing is not applicable to this article.

## Abbreviations

The following abbreviations are used in this manuscript:

EPS	Extracellular Polymeric Substances
SCFA	Short-Chain Fatty Acids
pH	Potential of Hydrogen
qPCR	Quantitative Real-Time Polymerase Chain Reaction
*S. mutans*	*Streptococcus mutans*
